# Knowledge, attitude, and practice of artificial intelligence in emergency and trauma surgery, the ARIES project: an international web-based survey

**DOI:** 10.1186/s13017-022-00413-3

**Published:** 2022-02-10

**Authors:** Belinda De Simone, Fikri M. Abu-Zidan, Andrew A. Gumbs, Elie Chouillard, Salomone Di Saverio, Massimo Sartelli, Federico Coccolini, Luca Ansaloni, Toby Collins, Yoram Kluger, Ernest E. Moore, Andrej Litvin, Ari Leppaniemi, Pietro Mascagni, Luca Milone, Micaela Piccoli, Mohamed Abu-Hilal, Michael Sugrue, Walter L. Biffl, Fausto Catena

**Affiliations:** 1 Department of Emergency and Metabolic Minimally Invasive Surgery, Centre Hospitalier Intercommunal de Poissy/Saint Germain en Laye, 10 Rue de Champ Gaillard, Poissy Cedex, France; 2 Department of Surgery, College of Medicine and Health Sciences, UAE University, Al-Ain, United Arab Emirates; 3 Department of General Surgery, Ospedale Civile “Madonna del Soccorso”, San Benedetto del Tronto, AP Italy; 4 Department of General Surgery, Macerata Hospital, Macerata, Italy; 5 Department of Surgery, Pisa University Hospital, Pisa, Italy; 6 Department of General Surgery, University Hospital of Pavia, Pavia, Italy; 7 IRCAD, Strasbourg, France; 8 Department of Emergency and Trauma Surgery, Rambam Health Campus, Haifa, Israel; 9 Department of Surgery, School of Medicine and the Ernest E. Moore Shock Trauma Center at Denver Health, University of Colorado, Denver, CO USA; 10 Abdominal Center, Helsinki University Hospital and University of Helsinki, Helsinki, Finland; 11 Department of Surgical Disciplines, Immanuel Kant Baltic Federal University, Regional Clinical Hospital, Kaliningrad, Russia; 12 Fondazione Policlinico Universitario A. Gemelli IRCCS, Rome, Italy; 13 Department of General and Robotic Surgery, The Brooklyn Hospital Center, New York, USA; 14 Division of General, Emergency Surgery and New Technologies, Ospedale Civile Di Baggiovara, Azienda Ospedaliero - Universitaria Di Modena, Modena, Italy; 15 Hepato-Bilio-Pancreatic Minimally Invasive Surgery, Poliambulanza Foundation Hospital, Brescia, Italy; 16 Department of Surgery, Letterkenny University Hospital Ireland, Letterkenny, Ireland; 17 Department of Trauma and Acute Care Surgery, Scripps Memorial Hospital, La Jolla, CA USA; 18 Department of Emergency and Trauma Surgery, Bufalini Hospital, Cesena, Italy

**Keywords:** Artificial intelligence, Emergency surgery, Trauma surgery, Research, Survey, Decision making, Robotic surgery, Laparoscopy

## Abstract

**Aim:**

We aimed to evaluate the knowledge, attitude, and practices in the application of AI in the emergency setting among international acute care and emergency surgeons.

**Methods:**

An online questionnaire composed of 30 multiple choice and open-ended questions was sent to the members of the World Society of Emergency Surgery between 29th May and 28th August 2021. The questionnaire was developed by a panel of 11 international experts and approved by the WSES steering committee.

**Results:**

200 participants answered the survey, 32 were females (16%). 172 (86%) surgeons thought that AI will improve acute care surgery. Fifty surgeons (25%) were trained, robotic surgeons and can perform it. Only 19 (9.5%) were currently performing it. 126 (63%) surgeons do not have a robotic system in their institution, and for those who have it, it was mainly used for elective surgery. Only 100 surgeons (50%) were able to define different AI terminology. Participants thought that AI is useful to support training and education (61.5%), perioperative decision making (59.5%), and surgical vision (53%) in emergency surgery. There was no statistically significant difference between males and females in ability, interest in training or expectations of AI (*p* values 0.91, 0.82, and 0.28, respectively, Mann–Whitney U test). Ability was significantly correlated with interest and expectations (*p* < 0.0001 Pearson rank correlation, rho 0.42 and 0.47, respectively) but not with experience (*p* = 0.9, rho − 0.01).

**Conclusions:**

The implementation of artificial intelligence in the emergency and trauma setting is still in an early phase. The support of emergency and trauma surgeons is essential for the progress of AI in their setting which can be augmented by proper research and training programs in this area.

**Supplementary Information:**

The online version contains supplementary material available at 10.1186/s13017-022-00413-3.

## Background

Artificial Intelligence (AI) is defined as the study of algorithms that give machines the ability to perform human-like tasks and perform cognitive functions that they were not necessarily programmed for such as problem-solving, object and word recognition, and decision-making [1]. It is a very complex branch of computer engineering that covers various fields of research including machine learning, natural language processing, artificial neural networks, and computer vision [main definitions are summarised in Additional file 1: Appendix 1]. It can be applied in the daily practice of medicine and has had an exponential increase in interest over the last few years. It is gradually supporting surgical practice using technological advancements in imaging, patient management navigation, and robotic interventions [2]. Surgery is associated with AI in robotic surgery which is a novel evolution of minimally invasive surgery aiming to improve surgical outcomes and patient’s experience [3].

To our knowledge, there are few articles focused on the utility of AI technologies in emergency and trauma surgery specifically [4–7]. Emergency surgeons take decisions that have major impacts on patients. This needs an accurate assessment of the patient's clinical and radiological data to have a favourable clinical outcome. Accordingly, AI can be very useful in these highly demanding, stressful, and serious situations. The **Ar**tificial **I**ntelligence in **E**mergency and Trauma **S**urgery (ARIES) survey aimed to evaluate the knowledge, attitude, and practices in the application of AI in the emergency setting among international acute care and emergency surgeons.

## Methods

### Ethical considerations

This survey evaluated the perception, attitude, and knowledge of emergency and trauma surgeons on the use and application of AI in emergency and trauma surgery. Participation in the survey was voluntary, and the participants’ data were anonymized. No personally identifiable data were collected. Accordingly, ethical approval was not required.

### Study design

A cross-sectional study among the members of the World Society of Emergency Surgery (WSES) between 29th May and 28th August 2021 (3 months period).

### Sample size

We have sent the questionnaire to 10,000 mailing list’s members of the World Society of Emergency Surgery (WSES). Accordingly, sample size calculation was not needed. The response rate was 2% (200/10000).

### Questionnaire design

The main objectives of the survey were to (1) assess the interest of acute care surgeons in AI application in the management of acute care surgical patients and to (2) define priorities of research projects in the implementation of AI in trauma and acute care surgery.

This online questionnaire was composed of 30 multiple choice and open questions (Additional file 1: Appendix 1) and was designed following the Checklist for Reporting Results of Internet E-Surveys (CHERRIES) [8–11]. The questionnaire was divided into 5 sections: (1) Demographic: questions (1–8), (2) Skill and technology questions (9–16), (3) AI knowledge questions (17–19), (4) Expectations and involvement questions (20–27), and (5) Suggestions questions (28 to 30).

The questionnaire was initially written by BDS. It was then sent to an international expert panel consisting of 10 members for their advice and modifications including members of the Editorial Board of the Artificial Intelligence Surgery Journal, especially of PM and TC, authors of a previous survey about AI [https://forms.gle/hHeyEv3EE3Ff3Vcm7], and AG who gave guidance on the questions regarding autonomous actions. Finally, the definitive questionnaire was submitted to the WSES steering committee to be endorsed.

### Validity and piloting

We depended mainly on surface validity for validation while content validity depended mainly on the experts’ experience in this area. We did not pilot the questionnaire for linguistic clarity because it was reviewed by 9 international experts who stemmed from 5 different countries speaking different mother tongue languages including English, Arabic, French, Italian, and Russian assuring the clarity of the questionnaire for the international participants.

### Distribution of the survey and data collection

The online survey was built upon a google form platform which was accessed through the WSES website. The survey was sent by mail to the WSES members through the mailing list. Data were collected and stored in an online database protected by a password known only by the principal investigator. The survey was announced, advertised, and diffused by the WSES website with a programmed invitation to participate sent by mail, for 3 months (29th May to 28th August 2021).

### Statistical analysis

Statistical analysis was carried out using non-parametric methods. Mann Whitney U test was used to compare two independent groups. Kruskal–Wallis test was used to compare three independent groups while Pearson rank correlation was used to correlate different ranks. A p-value of less than 0.05 was considered significant.

## Results

Two hundred participants answered the survey. Of these, 134 (67%) work in academic centers, 58 (20%) in community hospitals, and six (3%) in private clinics. One hundred twenty-nine (64%) were consultants, 42 (21%) were attending surgeons, and 29 (14%) were residents. Sixty-two (31%) surgeons have a work experience of 11–20 years, 55 (27%) of 5–10 years, 39 (19%) of less than 5 years, 23 (11%) more than 30 years, and 21(10%) of 21–30 years. Only 32 were females (16%). One hundred seventy-two (86%) surgeons were confident that AI will improve their acute care surgery practice, 17 (8.5%) thought that it will not affect their job, while 6 thought that it will make it more difficult (3%).

Seventy-five surgeons (37.5%) perform minimally invasive surgery in 51–75% of their procedures, 44 (22%) in 25–50% of their procedures, 38 (19%) in 76–90% of their procedures, 24 (12%) in less than 25% of their procedures, 12 (6%) (12/200) in more than 90% of their procedures while seven surgeons (3.5%) do not perform minimally invasive surgery. Minimally invasive surgery was performed in both elective and emergency surgery by 149 surgeons (74.5%), only in elective surgery by 39 (19%) surgeons, and only in emergency surgery by 8 surgeons (4%). Fifty surgeons (25%) were trained, robotic surgeons. Only 19 (9.5%) were currently performing robotic surgery.

**Fig. 1:**
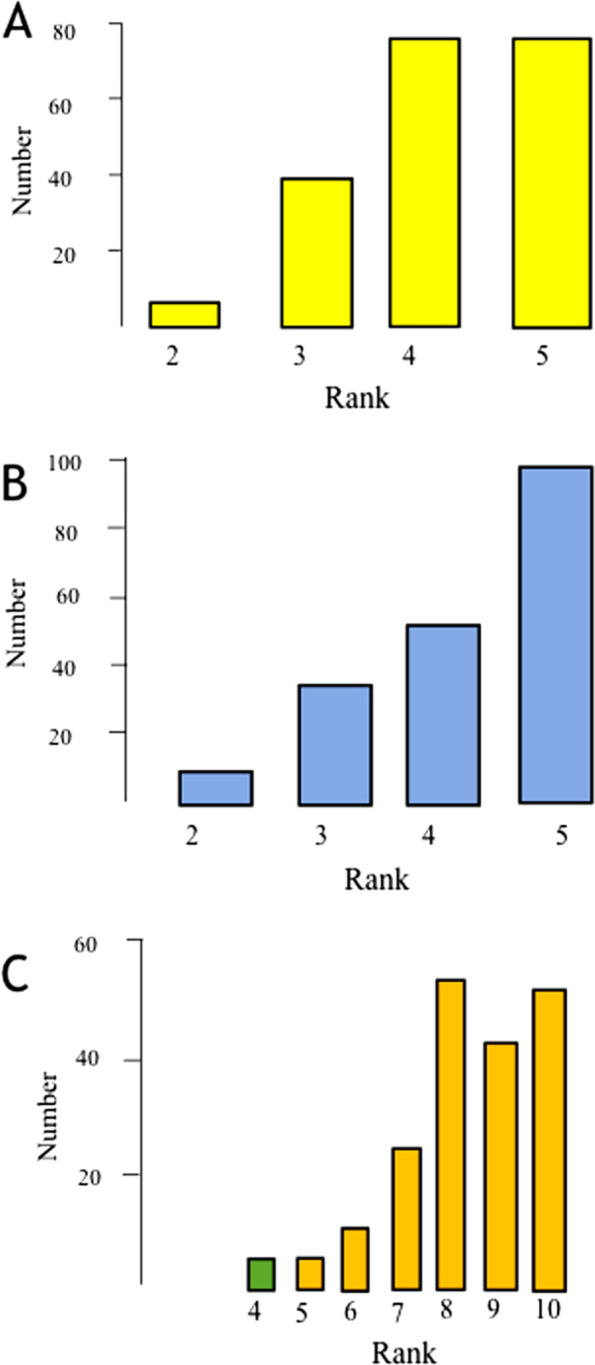
Expectations, interest, and abilities of the respondents where **a** is the answer for “Do you think that Artificial intelligence can improve emergency surgery” on a scale (rank) of 5 where 1 is not at all and 5 is extremely”; **b** is the answer for the question “ What is your interest in a course or research emergency surgery on a scale (rank) of 5 where 1 not at all and 5 is extremely” and **c** is the answer for the question “What is your ability in adopting new technologies on a scale (ranks) of 1–10 where 1 is very slow while 10 in an enthusiastic adopter of new technology”

One hundred twenty-six (63%) surgeons do not have a robotic system in their institution, and for those who have it, it was mainly used for elective surgery. One hundred ten surgeons (55%) have experience in the 3D system of vision which was mainly in elective surgery. Only 100 surgeons (50%) were able to define different AI terminologies like general and narrow AI, machine and deep learning, supervised and unsupervised learning, computer vision and natural language processing. Seventy-seven surgeons (38.5%) read AI-based surgical articles and feel comfortable with their details, whereas 56 (28%) didn’t read articles about AI (Table 1, Additional file 2: Appendix 2). Seventy-seven (38.5%) surgeons think that AI can extremely improve emergency and trauma surgery, and 99 (49.5%) were highly interested in courses or research projects about the application of AI in emergency surgery (Fig. 1a, b). The majority of the participants thought that they are quick adopters for new technologies Fig. 1c.

**Table 1:** Emergency surgeons’ knowledge of artificial intelligence terms and publications, the usefulness of artificial intelligence in clinical practice for emergency and trauma surgeons, and research topics of interest

	I cannot define/ distinguish any of them (%)	I can define/ distinguish some of them (%)	I can define/distinguish all of them (%)	I am familiar with more advanced AI concepts than these (%)
**Knowledge of artificial intelligence**				
Are you familiar with the following Artificial Intelligence (AI) terms? General and narrow AI, machine and deep learning, supervised and unsupervised learning, computer vision and natural language processing	48 (23.8)	100 (49.5)	40 (19.8)	14 (6.9)

	No (%)	-I read AI-based surgical articles, but I find them confusing (%)	-I read AI-based surgical articles and I feel comfortable with their details (%)	I read AI-based computer science and engineering articles and find them confusing (%)	I read AI-based computer science and engineering articles and understand them (%)
Did you read scientific articles on artificial intelligence (AI)?	56/200 (28)	39/200 (19.5)	77/200 (38.5)	6/200 (3)	22/200 (11)

	Perioperative decision making (%)	Intraoperative decision making (%)	Improved surgical vision (%)	Surgical practice (%)	Training and education (%)	Surgical robot automation (%)	High technology devices for surgery (%)
**Usefulness of artificial intelligence**							
I think artificial intelligence applications in emergency surgery are useful for	115 (57.5)	84 (42)	107(53.5)	100 (50)	120 (60)	–	–
I think important artificial intelligence research areas in emergency surgery should include	119(59.5)	94(47)	106(53)	97(48.5)	123(61.5)	57(28.5)	103(51.5)

The majority of surgeons believe that AI in emergency surgery can be useful to support peri-operative decision making, improved surgical vision, surgical practice, training, and education (Table 1). The highest areas were training and education (61.5%), perioperative decision making (59.5%), and improved surgical vision (53%), (Table 1). 93% of the surgeons thought that high technologies such as the da Vinci System, I-Drive, and Ligasure should be included in future research. The majority (93%) of the surgeons want to be involved in future research. Additional file 3: Appendix 3 summarises the suggested research topics by the participants.

Seventy-nine surgeons (39.5%) systematically collect clinical data in their practice (videos, images, and databases), 61 (30.5%) occasionally collect data (videos of surgical procedures), and 39 (19.5%) collected data by official requests. The majority of surgeons (149, 74.5%) were confident that AI will be available in their setting in the future.

There was no statistically significant difference between males and females in ability, interest in training and expectations of AI (*p* values 0.91, 0.82, and 0.28, respectively, Mann–Whitney U test).

There was also no statistically significant difference between residents, attending surgeons and consultants in ability, interest in training and expectations of AI (*p* values 0.82, 0.82, and 0.93, respectively, Kruskal–Wallis test). Ability was significantly correlated with interest and expectations (*p* < 0.0001 Pearson rank correlation, rho 0.42 and 0.47, respectively) but not with experience (*p* = 0.9, rho − 0.01) (Table 2).

**Table 2:** Correlations between surgical experience, ability, interest, and expectations of artificial intelligence; Rho and *p* value of Spearman rank correlation

	Statistics	Surgical experience	Ability	Interest	Expectations
Surgical experience	Rho	–	− 0.01	− 0.11	− 0.06
	*p* value	–	0.9	0.11	0.42
Ability	Rho	− 0.01	–	0.42	0.47
	*p* value	0.9	–	< 0.0001	< 0.0001
Interest	Rho	− 0.11	0.42	–	0.66
	*p* value	0.11	< 0.0001	–	< 0.0001
Expectations	Rho	− 0.06	0.47	0.66	–
	*p* value	0.42	< 0.0001	< 0.0001	–

## Discussion

Our study has shown that the majority of our participants perform minimally invasive surgery in emergency surgery. Nevertheless, only 25% are trained to perform robotic surgery and less than 10% currently practice it which highlights the need for more training in this area. Furthermore, more than 60% of the participants do not have a robotic system in their hospitals. Females have shown similar interest in AI in emergency and trauma surgery to males.

The application of innovative technologies can improve decision making and surgical outcomes. This usually follows an S-shaped curve having 3 phases: (1) the introduction phase, (2) the performance improvement phase, and finally (3) the plateau phase. This is followed by augmentation of its use by replacement of existing standards [3, 12, 13]. Robotic surgery is an evolution of laparoscopic surgery which has a high magnification of the 3-dimensional views, reduction or elimination of hand tremors, instruments that articulate to extreme angles, and comfortable ergonomics and platforms [14]. Several studies have shown that robotic surgery is safe and feasible in the emergency setting. The main obstacle to its adoption is the lack of training and accessibility [15–17]. The current study clearly shows that trauma and acute care surgeons are enthusiasts and supportive of the use of AI in their clinical practice.

The participants supported research in autonomous actions in surgery [18]. Intelligent technologies may improve vision by using 3D systems, “feel” the thickness of tissues to cut them (using surgical staples) or create vessel fusion (by adjusting energy delivery to the tissue). These actions are achieved by using mathematical algorithms which determine the right action, at the right time, with minimal human supervision.

The majority of participants thought that AI tools are useful in supporting decision making, which may improve clinical outcomes. Critical decision making in the acute care setting is a complex individual clinical reasoning cognitive process affected by emotions, limited time, lack of information, and high risk. Loftus et al. [19] showed that traditional clinical-decision support systems are time-consuming because of manual data management and do not consider the nonlinear relationship among multiple non-static variables, decreasing accuracy. AI models, fed with live streaming intraoperative and electronic health record data, integrated with bedside assessment and human intuition could improve critical decision-making.

For example, in performing a safe laparoscopic cholecystectomy, the fulfilment of the criteria of critical view safety (CVS) are required to prevent bile duct injuries, despite that a bile duct injury can occur in managing severe cholecystitis. Deep learning models, built using high quality video-reporting datasets, could assist emergency surgeons in intra-operative decision making, in performing a safe laparoscopic cholecystectomy [20]. In practice, it means that CVS is assessed using computer vision, and the anatomy will be segmented on the operating room screen in safe and dangerous (NO-GO) zones of dissection, liver, gallbladder, and hepatocystic triangle during laparoscopic cholecystectomy, to decrease intraoperative errors in visual perception and judgment leading to misinterpretation of anatomy.[21].

The implementation of decision support systems based on Artificial Neural Networks is limited by: (1) the quality and reliability of medical information, (2) the lack of transparency in the decision-making process, (3) the selection and development of personnel capable of effectively using and maintaining intelligent systems, (4)the high cost and (5) and finally security issues [22].

Large international secured databases may facilitate the development of highly reliable and accurate AI algorithms. It is important to acknowledge that the WSES was very supportive of this issue by developing surgical registers to collect large-high-quality international data [23]. Participating in these international databases with high-quality data will improve the development of useful AI tools. Nevertheless, errors may occur when developing AI algorithms. Accountability for these errors poses an important ethical dilemma. O’Sullivan et al. [24] classified responsibility in autonomous robotic surgical procedures into accountability, liability, and culpability. Supervision of the treating surgeon during the early phase of AI implementation is highly important. Other legal issues include privacy, cybercrime, following ethical standards, and human representation [25].

**Fig. 2:**
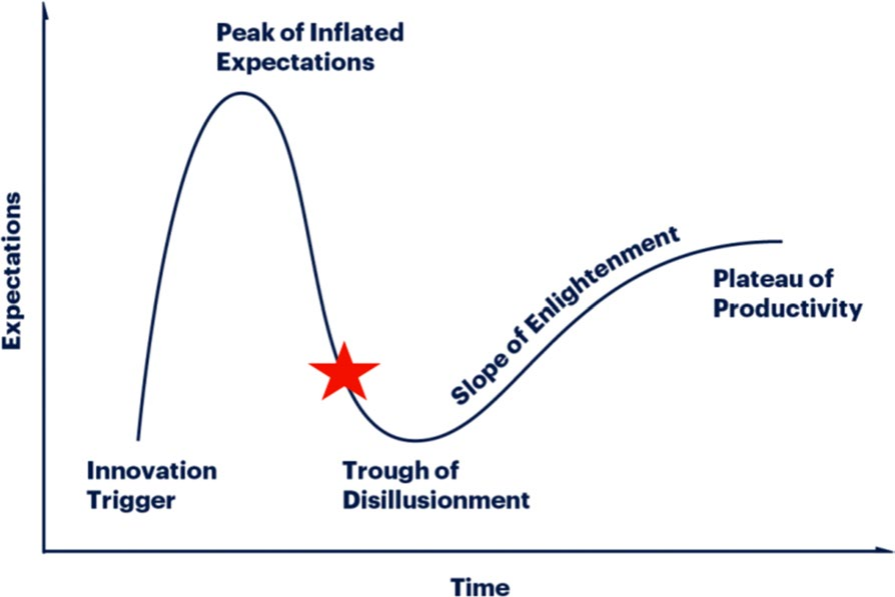
The Gartner Hype Cycle methodology describes how the perceived value of a given technology evolves

The Gartner Hype Cycle methodology [26] describes how the perceived value of a given new technology evolves in several phases (Fig. 2): the initial enthusiasm phase, the mass adoption and evaluation phase, and finally the maturity phase after technology improvement. The limited access of trauma and emergency surgeons to robotic surgery due to unavailability, high cost, and lack of training have delayed its implementation in clinical practice. We think that AI applications will improve emergency surgery outcomes. The current survey shows that the ability in adopting new technologies is significantly correlated with interest and expectations.

## Limitations

This study has certain limitations. *First,* the response rate is low, and the sample size is relatively small. This increase the risk of selection bias. Those who are interested in AI may have responded with a relatively higher percentage reported in those being trained in AI. This may not reflect reality. *Second,* this study is survey-based which depends on subjective opinions and carries the risk of recall bias. *Third,* we depended on surface validity and expert opinion in developing this questionnaire. Nevertheless, we think that the information we gained is useful and helps guide us in exploring this important area.

## Conclusions

The ARIES survey showed that the implementation of AI in the emergency and trauma setting is still in an early phase, despite the high interest showed by emergency surgeons invited to join the ARIES project. Its progression needs focusing research in the most useful fields of interest to improve patients’ outcomes. Emergency and trauma surgeons claimed to be involved. The support of emergency and trauma surgeons by health care systems and industries is essential for the progress of AI in their setting which will be augmented by proper research and training programs in this area.

## Supplementary Information

**Additional file 1. Appendix 1:** ARIES survey form.

**Additional file 2. Appendix 2:** Some Artificial Intelligence definitions [2-8-14].

**Additional file 3. Appendix 3:** Research topics suggested from ARIES participants.

**Additional file 4. Appendix 4:** ARIES collaborative group.

## Data Availability

Not applicable.

## Abbreviations

AI: Artificial Intelligence
WSES: World Society of Emergency Surgery
ARIES: **Ar**tificial **i**ntelligence in **e**mergency and trauma **s**urgery
ES: Emergency surgery
