# Supplementary material for: Knowledge, attitude, and practice of artificial intelligence in emergency and trauma surgery, the ARIES project: an international web-based survey
Source: World J Emerg Surg. 2022 Feb 10;17:10. doi: 10.1186/s13017-022-00413-3 (PMC8832812; doi:10.1186/s13017-022-00413-3)
Supplement: Supplementary file 1 — Additional file 1. Appendix 1: ARIES survey form. [file 13017_2022_413_MOESM1_ESM.pdf]

ARtificial Intelligence in Emergency and trauma Surgery (ARIES) project: WSES-WJES survey in joint venture with AISJ

Dear Colleague,

Artificial intelligence (AI) and its applications in everyday life and medicine have been increasing exponentially over the last several years.

In surgery, it is common to associate AI with robotic surgery, which represents an evolution of minimally invasive surgery, but it is not only this!

AI is a very complex branch of computer engineering that covers various fields of research (i.e. machine learning, natural language processing, artificial neural networks and computer vision, to name a few).

In reviewing the literature, the utility of artificial intelligence technologies in acute care surgery remains unexplored.

We know well that in an emergency situation the ability to make quick decisions and an accurate assessment of the patient's clinical and radiological data is the key to decreasing mortality and morbidity rates and AI could be very useful in this setting.

This survey was conceived to assess the interest among international acute care surgeons to discuss aspects of AI applied to the management of patients in the emergency setting.

Thank you for participating.

P.I.: Belinda De Simone, MD
Centre Hospitalier Intercommunal de Poissy et St Germain en Laye (France)

Expert Panel
Mohammed Abu Hilal (Poliambulanza Brescia-Italy)
Fikri M. Abu-Zidan, (Al-Ain, United Arab Emirates (research methodologist)
Walter Biffl (Scripps Memorial Hospital La Jolla, La Jolla, California-USA)
Elie Chouillard (Centre Hospitalier Intercommunal de Poissy/Saint Germain en Laye-France)
Andrew Gumbs (Centre Hospitalier Intercommunal de Poissy/Saint Germain en Laye-France; Editor in Chief Artificial Intelligence Surgery Journal)
Pietro Mascagni (Fondazione Policlinico Universitario A. Gemelli IRCCS, Rome, Italy)
Luca Milone (Brooklyn Hospital Center-USA)
Andrej Litvin (Immanuel Kant Baltic Federal University, Regional Clinical Hospital Kaliningrad, Russia)
Micaela Piccoli (AOU di Modena-Italy)
Toby Collins (IRCAD)

WSES Steering Committee
Fausto Catena (Cesena, Italy)
Massimo Sartelli (Macerata, Italy)
Salomone Di Saverio (Varese, Italy)
Luca Ansaloni (Pavia, Italy)
Micheal Sugrue (Dublin, Ireland)
Yoram Kluger (Rambam, Israel)
Ernest E Moore (USA)
Federico Coccolini (Pisa, Italy)
Ari Leppaniemi (Finland)

---

*Required

1. Email *

____

2. Country of work *

____

3. Affiliation

____

4. Gender *

Mark only one oval.

- ◯ female
- ◯ male

5. Main hospital setting *

Mark only one oval.

- Private practice
- Community hospital
- Academic center
- other

6. Surgical Specialty *

____

7. Hospital Position *

Mark only one oval.

- Consultant
- Medical student
- Attending surgeon
- Resident

8. Surgical experience (years) *

Mark only one oval.

- <5
- 5-10
- 11-20
- 21-30
- >30

9. What's your ability of adopting new technologies on a scale of 1 to 10 where 1 =very slow adopter and 10=enthusiastic developer of new technologies? *

Mark only one oval.

- 1
- 2
- 3
- 4
- 5
- 6
- 7
- 8
- 9
- 10

10. What percentage does minimally invasive surgery constitute out of your surgical practice? *

Mark only one oval.

- none
- <25%
- 25-50%
- 51-75%
- 76-90%
- >90%

11. What areas do you perform minimally invasive surgery in? *

Mark only one oval.

- elective surgery
- emergency surgery
- both
- no one

12. Are you trained and can perform robotic surgery? *

Mark only one oval.

- yes
- no

13. Do you currently perform robotic surgery? *

Mark only one oval.

- Yes
- No

14. Is there a robotic system available in your institution? *

Mark only one oval.

- Yes, only for elective surgery
- Yes for elective and emergency surgery
- No

15. Do you have experience with a 3D system of vision? *

Mark only one oval.

- Yes
- No

16. If yes: *

Mark only one oval.

- Only for scheduled surgical procedures
- Only for surgical oncology
- Other: ______________________

17. Are you familiar with the following Artificial Intelligence (AI) terms? General and narrow AI, machin and deep learning, supervised and unsupervised learning, computer vision and natural language processing. *

Mark only one oval.

- I cannot define/distinguish any of them
- I can define/distinguish some of them
- I can define/distinguish all of them
- I am familiar with more advanced AI concepts than these

18. Did you read scientific articles on artificial intelligence (AI)? *

Mark only one oval.

- No
- -I read AI-based surgical articles, but I find them confusing
- -I read AI-based surgical articles and I feel comfortable with their details
- I read AI-based computer science and engineering articles and find them confusing
- I read AI-based computer science and engineering articles and understand them

19. What is your interest in course or research about the application of AI in emergency surgery on a scale 1 to 5 where 1=not interested and 5=highly interested *

Mark only one oval.

- 1
- 2
- 3
- 4
- 5

20. Do you think that artificial intelligence can improve emergency and trauma surgery on a scale of 1 to 5 where 1=not at all and 5=extremely? *

Mark only one oval.

- 1
- 2
- 3
- 4
- 5

21. I think artificial intelligence applications in emergency surgery are useful for: *

Tick all that apply.

- [ ] perioperative decision making
- [ ] intraoperative decision making
- [ ] improved surgical vision
- [ ] surgical practice
- [ ] training and education

22. I think important artificial intelligence research areas in emergency surgery should include: *

Tick all that apply.

- [ ] perioperative decision making
- [ ] intraoperative decision making
- [ ] surgical robot automation
- [ ] surgical procedures
- [ ] improved surgical vision
- [ ] training and education
- [ ] high technology devices for surgery

23. Do you think that technologies, i.e Da Vinci system, I-Drive and LigaSure, should be included in a research project related to AI in surgery? *

Mark only one oval.

- ( ) Yes
- ( ) No

24. Do you collect surgical data? *

Mark only one oval.

- No
- Only within official/requested protocols
- Occasionally record videos of surgical procedures
- Systematically record videos of surgical procedures
- Systematically collect database data including surgical videos and images

25. I think that the study of autonomous actions during acute surgery: *

Mark only one oval.

- should be prioritized during the initial phase of study
- should be minimized during the initial phase of study
- I'm not sure

26. Do you think AI will impact your job as an acute care surgeon? *

Mark only one oval.

- yes, it will improve my job
- No, it will not affect my job
- yes, it will make my job more difficult
- yes, it may completely replace me

27. What is the possibility that your institution will implement artificial intelligence applications? *

Mark only one oval.

- Almost none
- Possibly in the future
- Immediately partially
- Immediately full

28. Do you have some suggestions for further research in implementation of artificial intelligence in emergency and trauma surgery?

29. Do you want to be involved in research about AI and emergency and trauma surgery? *

Mark only one oval.

- No
- Yes

30. if yes, write your mail here

This content is neither created nor endorsed by Google.

Google Forms
