# Supplementary material for: Knowledge, attitude, and practice of artificial intelligence in emergency and trauma surgery, the ARIES project: an international web-based survey
Source: World J Emerg Surg. 2022 Feb 10;17:10. doi: 10.1186/s13017-022-00413-3 (PMC8832812; doi:10.1186/s13017-022-00413-3)
Supplement: Supplementary file 2 — Additional file 2. Appendix 2: Some Artificial Intelligence definitions [2-8-14]. [file 13017_2022_413_MOESM2_ESM.docx]

**Appendix 2:** Some Artificial Intelligence definitions [2-8-14]

**Machine learning (ML)** enables machines to learn and make predictions by recognizing patterns. ML allows a computer to utilise partial labelling of the data (supervised learning) or the structure detected in the data itself (unsupervised learning) to explain or make predictions about the data without explicit programming. Supervised learning is useful for training a ML algorithm to predict a known result or outcome while unsupervised learning is useful in searching for patterns within data.

**Deep Learning (DL):** is a subfield of ML in which computer systems learn and represent highly dimensional data by adjusting weighted associations among input variables across a layered hierarchy of neurons or artificial neural network. Deep learning networks are neural networks comprised of many layers and are able to learn more complex and subtle patterns than simple one or two-layer neural networks. In deep models, the initial input and final output layers are connected by hidden layers containing hidden nodes, Each hidden node is assigned a weight that is influenced by previous layers, affects the output from that neutron, and has the potential to affect the outcome classification of the entire network. An algorithm optimises and updates weights as the model is trained to achieve the strongest possible association between input and output layers.

**Reinforcement learning (RL):** is an AI subfield in which computer systems identify actions yielding the highest probability of an outcome that is an agent learns that specific actions under certain conditions lead to rewards and penalties, using this knowledge to identify actions that achieve an ultimate goal. Two characteristics distinguish reinforcement learning from ML:1) trial-and-error search to identify the best action and 2) delayed reward, that is choosing actions that achieve the ultimate goal rather than short-term rewards.

**Natural language processing (NLP)** is a subfield of AI that emphasises building a computer’s ability to understand human language and is crucial for large scale analyses of content such as electronic medical record data, especially physicians’ narrative documentation. To achieve human-level understanding of language, successful NLP systems must expand beyond simple word recognition to incorporate semantics and syntax into their analyses.

**Artificial neural networks (ANN): is** a subfield of ML; ANN are inspired by biological nervous systems and have become of paramount importance in many AI applications. Neural networks process signals in layers of simple computational units (neurons); connections between neurons are then parameterized via weights that change as the network learns different input-output maps corresponding to tasks such as pattern/image recognition and data classification.

**Computer vision** describes machine understanding of images and videos, and significant advances have resulted in machines achieving human-level capabilities in areas such as object and scene recognition. Important healthcare-related work in computer vision includes image acquisition and interpretation in axial imaging with applications including computer-aided diagnosis, image-guided surgery, and virtual colonoscopy. Initially influenced by statistical signal processing, the field has recently shifted significantly towards more data-intensive ML approaches, such as neural networks, with adaptation into new applications.
