# Supplementary material for: Knowledge, attitude, and practice of artificial intelligence in emergency and trauma surgery, the ARIES project: an international web-based survey
Source: World J Emerg Surg. 2022 Feb 10;17:10. doi: 10.1186/s13017-022-00413-3 (PMC8832812; doi:10.1186/s13017-022-00413-3)
Supplement: Supplementary file 3 — Additional file 3. Appendix 3: Research topics suggested from ARIES participants. [file 13017_2022_413_MOESM3_ESM.docx]

**Appendix 3:** Research topics suggested from ARIES participants

**Do you have some suggestions for further research in implementation of artificial intelligence in emergency and trauma surgery?**

Radiomics in emergency and trauma surgery

Decision support systems based on artificial neural networks in emergency surgery

Combination of blockchain technology and artificial intelligence for emergency surgery tasks"

Usage in predication, prognosis, complication risk of emergency surgery

perioperative decision making, predicting complications, predicting complications, surgical procedures, training and education,

Decision making; training

We need to do a little more work to quantify human intelligence so that we understand what we really need from artificial intelligence

Machine learning or deep learning systems in perioperative decision making

Big data on laboratory markers can aid in the diagnosis and management plan of many diseases, including acute appendicitis, acute diverticulitis, mesenteric ischemia etc.

Yes , we are very interested but we are at far stage, I am studying python programming, deep learning and furthe

I am working on it, it is part of my field of research on surgery in space missions and I think that improvement in knowledge are needed

The possibility of predicting higher mortality risks with diagnosis may encourage more systematic evaluation of patients and perhaps earlier referral to experienced centers.

education of young surgeons

Integration into current practice flow will be paramount

Artificial intelligence should include perioperative and intraoperative imaging. Then Surgical Interventions can be randomly studied.

Multi centre study

can explore in prediction, survival and prognosis factor with AI in emergency and trauma surgery

development of preoperative scores and algorithms for surgical pathology

Decision making Algorithms

Create a better and earlier score than ISS

Use of 3D

Improvement in the preoperative assessment of trauma patients and the correct application and use of trauma scores and algorithms

I think that you should offer an online course on Artificial Intelligence in emergency surgery. This would improve research expectations"

I am currently working on the development of a protocol for machine learning in surgery in space missions, for now it is only an initial phase

AI in hyperspectral imaging to enhance intraoperative finding
