# Supplementary material for: Knowledge, attitude, and practice of artificial intelligence in emergency and trauma surgery, the ARIES project: an international web-based survey
Source: World J Emerg Surg. 2022 Feb 10;17:10. doi: 10.1186/s13017-022-00413-3 (PMC8832812; doi:10.1186/s13017-022-00413-3)
Supplement: Supplementary file 4 — Additional file 4. Appendix 4: ARIES collaborative group. [file 13017_2022_413_MOESM4_ESM.docx]

**ARIES collaborative group**

1. gerrymartines@virgilio.it
2. wspmrowe@hotmail.com
3. selmysabry2097@yahoo.com
4. kararda@yahoo.com
5. kujali2@gmail.com
6. lorenzo.cobianchi@unipv.it
7. negoiionut@gmail.com
8. yasinkara32@windowslive.com
9. gipe1984@gmail.com
10. elie.chouillard@ght-yvelinesnord.fr
11. maciej.waledziak@gmail.com
12. koky@brunet.bn
13. asschneck@yahoo.com
14. ahkink@gmail.com
15. lovenishbains@gmail.com
16. telonakos@hotmail.com
17. mauropodda@ymail.com
18. andreapicchetto@gmail.com
19. Veroux@unict.it
20. francesco.forfori@unipi.it
21. giuseppe.brisinda@policlinicogemelli.it
22. marina.troian@asugi.sanita.fvg.it
23. claudiacristina.lopesmoreira@osakidetza.eus
24. mihail_slavchev@yahoo.com
25. salomone.disaverio@gmail.com
26. aalitvin39@gmail.com
27. noushif@gmail.com
28. justin.davies@addenbrookes.nhs.uk
29. stefanoro1970@gmail.com
30. biagiopicardi@gmail.com
31. caxiaogomes@gmail.br
32. vladimirkhokha@gmail.com
33. andee@usm.my
34. dr_smahmud@yahoo.com
35. asschneck@yahoo.com
36. ichadmigm@gmail.com
37. massalou.d@chu-nice.fr
38. dinuzzip@gmail.com
39. martin.reichert@chiru.med.uni-giessen.de
40. Tadeja.pintar@kclj.si
41. walt@biffl.com
42. ari.leppaniemi@hus.fi
43. mika.ukkonen@fimnet.fi
44. leandros93@hotmail.it
45. aagumbs@gmail.com
46. narkevichart@gmail.com
47. darkokraljik@gmail.com
48. leandros93@hotmail.it
49. roland.croner@med.ovgu.de
50. abuhilal9@gmail.com
51. edgardlozada2@gmail.com
52. aleix.martinez.perez@gmail.com
53. ciancidoc1@virgilio.it
54. cpugh@stanford.edu
55. babismed@gmail.com
56. gachabayovmahir@gmail.com
57. fabuzidan@uaeu.ac.ae
58. lucamilone@gmail.com
59. kobeyoshiro@gmail.com
60. kostastsekos@gmail.com
61. federico.coccolini@gmail.com
62. Marco.vito.marino@gmail.com
63. g.tomadze@tsmu.edu
64. augustin.goran@gmail.com
65. antonio.pesce@ausl.fe.it
66. teresaperra1992@gmail.com
67. alberto.porcu.1@gmail.com
68. drahmedsaeed@hotmail.com
69. marina.valente@unipr.it
70. adam.peckham-cooper@nhs.net
71. mike_truitt@hotmail.com
72. kebebeb21@gmail.com
73. drcalu@yahoo.com
74. dr.giorgiogiraudo@gmail.com
75. erythrokim@paik.ac.kr
76. massimosartelli@gmail.com
77. francesco_roscio@yahoo.it
78. lukas_widmer@bluewin.ch
79. nikolaospararas@yahoo.gr
80. desire.pantalone@unifi.it
81. marco.ceresoli89@gmail.com
82. hardcastle@ukzn.ac.za
83. rsydorchuk@bsmu.edu.ua
84. diego.sasia@hotmail.it
85. smrdemirli@hotmail.com
86. simone.garg27@gmail.com
87. ozmenmm@gmail.com
88. alconchelgago@gmail.com
89. musina.anamaria@gmail.com
90. veroux@unict.it
91. giuseppe.brisinda@policlinicogemelli.it
92. gianmaria.casonipattacini@gmail.com
93. efstratia.baili@gmail.com
94. tousur@hotmail.com
95. alessio.giordano8@gmail.com
96. Alessiovolpicelli94321@gmail.com
97. mohamed.elbahnaswy@med.tanta.edu.eg
98. majorpiotr@gmail.com
99. arpadpanyko@gmail.com
100. sandra_dios89@hotmail.com
101. tasisnikolaos@gmail.com
102. sofiabecks@yahoo.gr
103. mpapadoliop@outlook.com
104. virginia.dm.87@gmail.com
105. giuseppe.brisinda@policlinicogemelli.it
106. alfiejose@gmail.com
107. lcvdnc@unife.it
108. spirosgdelis@gmail.com
109. nmachair@gmail.com
110. fabrizioda@gmail.com
111. oknarfmulita@hotmail.com
112. emregonullu@yahoo.com
113. michele.ammendola@unicz.it
114. omeryalkin@gmail.com
115. domenicovita94@gmail.com
116. serhatmeric83@yahoo.com
117. lcvdnc@unife.it
118. amandashabana12@gmail.com
119. mauropodda@ymail.com
120. robert.sawyer@med.wmich.edu
121. akpal.jnmc@yahoo.com
122. zdemetr@yahoo.com
123. aitorlandaluceolavarria@gmail.com
124. yasinkara32@windowslive.com
125. ncillara@gmail.com
126. bonomoluca@gmail.com
127. mladenlucky@gmail.com
128. smrdemirli@hotmail.com
129. drmarinis@gmail.com
130. schizasad@gmail.com
131. diegovisconti81@gmail.com
132. hmousa@uaeu.ac.ae
133. oubaraket@gmail.com
134. mir87mb@libero.it
135. khoril@hotmail.com
136. emrahakin@sakarya.edu.tr
137. andee@usm.my
138. Boyko.Atanasov@mu-plovdiv.bg
139. giovanni.tebala@ouh.nhs.uk
140. e.lostoridis@gmail.com
141. luistallonaguilar@gmail.com
142. t_triantafilou@yahoo.com
143. virginia.dm.87@gmail.com
144. veroux@unict.it
145. dr.luigiconti@gmail.com
146. zulfubayhan@gmail.com
147. toma.adelina@gmail.com
148. esoctavian@gmail.com
149. ciancidoc1@virgilio.it
150. monikagureh33@gmail.com
151. andrea.balla@gmail.com
152. inikolopoulos@gmail.com
153. atoroct@gmail.com
154. franz.fleres@gmail.com
155. nmichal@ned.uoa.gr
156. andrea.bondurri@gmail.com
157. antodel88@libero.it
158. kararda@yahoo.com
159. drzeynepozkan@yahoo.com
160. edoardopoletto1992@gmail.com
161. sabino.capuzzolo@gmail.com
162. vladimirkhokha@gmail.com
163. m.malerba@asl2.liguria.it
164. dl.davideluppi@gmail.com
165. arcangelopicciariello@gmail.com
166. daniele.delogu@hotmail.com
167. mariarosaria.valenti@gmail.com
168. drviniciuscirurgia@icloud.com
169. roland.croner@med.ovgu.de
170. lovenishbains@gmail.com
171. Marcin.strzalka@uj.edu.pl
172. pant.greek@gmail.com
173. mesina.cristian@doctor.com
174. dott.g.soldini@gmail.com
175. guglielmo.clarizia@asst-val.it
176. a_spolini@yahoo.it
177. virginia.dm.87@gmail.com
178. leonardo.solaini@auslromagna.it
179. evgeni_d1984@yahoo.com
180. michaelm500@yahoo.com
181. sunildocv@gmail.com
182. schizasad@gmail.com
183. luis.pino@correounivalle.edu.co
184. hhaassll@gmail.com
185. gennaro.perrone82@gmail.com
186. omar.jibrel@gmail.com
187. kebebeb21@gmail.com
188. sa154cairo@hotmail.com
189. mujdatbalkan@yahoo.com
190. desire.pantalone@unifi.it
191. kdark7582@gmail.com
192. Iuliuco@gmail.com
193. atarasconi@gmail.com
194. victorywkong@yahoo.com
195. leandros93@hotmail.it
196. bsakakushev@gmail.com
197. ebaldini@inwind.it
198. habeebogundipe@gmail.com
